# Potassium transporter KUP9 participates in K^+^ distribution in roots and leaves under low K^+^ stress

**DOI:** 10.1007/s44154-022-00074-x

**Published:** 2022-12-12

**Authors:** Taro Yamanashi, Takeshi Uchiyama, Shunya Saito, Taiki Higashi, Hayato Ikeda, Hidetoshi Kikunaga, Mutsumi Yamagami, Yasuhiro Ishimaru, Nobuyuki Uozumi

**Affiliations:** 1 Department of Biomolecular Engineering, Graduate School of Engineering, Tohoku University, Sendai, 980-8579 Japan; 2 Research Center for Electron Photon Science, Tohoku University, Sendai, 980-0826 Japan; 3 Cyclotron and Radioisotope Center, Tohoku University, Sendai, 980-8578 Japan; 4 Institute for Environmental Sciences, Rokkasho, Kamikita, Aomori, 039-3212 Japan

**Keywords:** Potassium, KUP9, *Arabidopsis thaliana*, KUP/HAK/KT

## Abstract

Potassium (K) is a major essential element in plant cells, and KUP/HAK/KT-type K^+^ transporters participate in the absorption of K^+^ into roots and in the long-distance transport to above-ground parts. In *Arabidopsis thaliana*, KUP9 is involved in the transport of K^+^ and Cs^+^ in roots. In this study, we investigated KUP9 function in relation to the K^+^ status of the plant. The expression of *KUP9* was upregulated in older leaves on K^+^-depleted medium, compared to the expression of the other 12 KUP genes in the *KUP/HAK/KT* family in Arabidopsis. When grown on low K^+^ medium, the *kup9* mutant had reduced chlorophyll content in seedlings and chlorosis in older rosette leaves. Tissue-specific expression of *KUP9* determined by *KUP9* promoter:GUS assay depended on the K^+^ status of the plants: In K^+^ sufficient medium, *KUP9* was expressed in the leaf blade towards the leaf tip, whereas in K^+^ depleted medium expression was mainly found in the petioles. In accordance with this, K^+^ accumulated in the roots of *kup9* plants. The short-term ^43^K^+^ tracer measurement showed that ^43^K was transferred at a lower rate in roots and shoots of *kup9*, compared to the wild type. These data show that KUP9 participates in the distribution of K^+^ in leaves and K^+^ absorption in roots under low K^+^ conditions.

## Introduction

Potassium, an abundant cation and the major osmolyte in plants, is essential for growth and adaption to various biotic and abiotic stresses. Severe K^+^ deficiency causes reduction in growth and biomass, disruption of stomatal control, leaf chlorosis and subsequent loss of photosynthesis efficiency. Either as direct consequences of K^+^ deficiency or due to lack of some K^+^ dependent enzyme activity, K^+^-deficient plants are highly vulnerable to drought, salinity, high light and anoxia (Wang et al. 2013; Hasanuzzaman et al. 2018). Uptake of this indispensable nutrient and its translocation throughout the plant is achieved by families of K^+^ channels and transporters. The Arabidopsis genome contains several K^+^ specific transporter families, KUP/HAK/KT transporters, *Shaker* K^+^ channels (tetrameric channels carrying a voltage sensor domain), cation/proton antiporters (CPAs), and TPK-type K^+^ channels (Ahn et al. 2004; Gambale and Uozumi 2006; Gierth and Mäser 2007; Sharma et al. 2013; Tsujii et al. 2020). Specifically, the *Shaker* K^+^ channel AKT1 and the KT/KUP/HAK family member HAK5 are considered to be the main contributors to K^+^ import from the soil into root cells (Rubio et al. 2008, 2010; Pyo et al. 2010; Nieves-Cordones et al. 2019).

These two transport systems differ in their K^+^ transport affinity; HAK5 dominates root K^+^ uptake under submillimolar concentrations of external K^+^, whereas AKT1 is the major contributor at higher concentrations. Additionally, HAK5 is essential for K^+^ retrieval in K^+^ deprived saline environments because under those conditions, AKT1 promotes K^+^ leakage from roots (Nieves-Cordones et al. 2010). The transport activity of both HAK5 and AKT1 is regulated via phosphorylation by Calcineurin B-like protein CBL1 and CBL-interacting protein kinase CIPK23 (Xu et al. 2006; Lee et al. 2007; Ragel et al. 2015). This regulation may be related to the modulation of K^+^ uptake and translocation mediated by AKT1, HAK5 and SKOR (Nieves-Cordones et al. 2019).

The functional roles of *Shaker* K^+^ channels have been discussed in detail, such as regulation of stomatal movement, sugar retrieval from the phloem, and pollen tube development (Mouline et al. 2002; Hosy et al. 2003; Dreyer et al. 2017). In contrast, the functional role of KUP/HAK/KT proteins seems to be limited to K^+^ import and translocation. *Escherichia coli* Kup was the first member of the KUP/HAK/KT family to be identified*,* followed by the homologous HAK which was isolated from the eukaryote *Schwanniomyces occidentalis* (Schleyer and Bakker 1993; María et al. 1995)*.* These findings led to the isolation of homologs from plants (Quintero and Blatt 1997; Santa-maría et al. 1997; Fu and Luan 1998; Kim et al. 1998). Arabidopsis KUP1 and KUP2 were shown to import K^+^ into *E. coli* and Arabidopsis suspension cells (Kim et al. 1998). To study the transport activity of KT/KUP/HAK, a K^+^ uptake-deficient *E. coli* strain was used as a functional expression system, leading to confirmation of the K^+^ uptake activities of KUP 4, 5, 6, 7, 10 and 11(Uozumi 2001; Ahn et al. 2004). KUP2, whose expression is regulated by bHLH and WRKY transcription factors, participates in maintaining Na^+^/K^+^ homeostasis and confers salt tolerance in plants (Rajappa et al. 2020). Examination of a *kup2 kup6 kup8* triple mutant in Arabidopsis suggests that all three KUPs act as K^+^ effluxers in roots. In addition, *KUP6* expression was detected in guard cells and vascular tissues (Osakabe et al. 2013). KUP7 functions as a K^+^ uptake transporter *in planta*, based on its ability to transport K^+^ when expressed in yeast and on the sensitivity of the *kup7* mutant to K^+^ deficiency (Han et al. 2016). Surprisingly, KUP5 has cytosolic adenylate cyclase activity, which is essential for its K^+^ transport activity (Al-Younis et al. 2018). Recent publications described the significance of KUP8 in the prevention of heavy metal accumulation, and the involvement of KUP4/TRH1 in root hair formation and gravitropism (Sanz-Fernández et al. 2021; Templalexis et al. 2021).

Additionally, KT/KUP/HAK is known to be one of the main transporter families that mediate Cs^+^ import from the soil into plants, together with members of the cation/H^+^ exchanger (CHX) family or the cyclic nucleotide gated channel (CNGC) family (White and Broadley 2000; Kanter et al. 2010). Bacterial Kup transporters are different from K^+^ channels in terms of their tertiary structure (Sato et al. 2014; Tascón et al. 2020). *E. coli* Kup is permeable to Rb^+^ and Cs^+^ in addition to K^+^ and these ions can support cell proliferation during K^+^ deficiency (Tanudjaja et al. 2017). In contrast, in plants, no beneficial effects of Cs^+^ uptake have been reported, even though Cs^+^ also belongs to the Group I alkali metals and has similar chemical properties as K^+^ (Nieves-Cordones et al. 2017; Rai et al. 2017). Rather, Cs^+^ accumulation can cause severe growth retardation due to nutritional competition with K^+^ and blockage of certain K^+^ channels by Cs^+^. A number of studies have implicated HAK5 as the major contributor to Cs^+^ uptake into plants (Qi et al. 2008; Adams et al. 2013, 2019a; Alemán et al. 2014; Genies et al. 2017). In contrast, AKT1 is blocked by external Cs^+^, which leads to leakage of K^+^ from the roots under low K^+^ conditions and subsequently growth inhibition due to K^+^ deficiency (Adams et al. 2019a).

Recent reports provide evidence for a role of KUP9 in K^+^ acquisition, auxin homeostasis, IAA metabolism in root tips and negative regulation of Cs^+^ accumulation (Adams et al. 2019b; Zhang et al. 2020; Genies et al. 2021). The K^+^ and Cs^+^ uptake ability of KUP9 was previously established by *E. coli* assay (Kobayashi et al. 2010). But while *KUP9* expression is induced in response to low K^+^ condition, its subcellular localization is restricted to the ER (Genies et al. 2021). Thus, whether KUP9 directly mediates K^+^ import and Cs^+^ extrusion in vivo or affects these processes indirectly through another transport system in the plasma membrane remains unknown.

This study focused on the expression and function of Arabidopsis KUP9 during K^+^ deficiency and provides insight into additional physiological processes involved in regulation of *KUP9* expression. We observed *KUP9* expression in shoots and studied how loss of KUP9 activity in *kup9* mutant leads to chlorosis in rosette leaves under K^+^-depleted conditions. We therefore measured the expression of *KUP9* and K^+^ content and performed imaging of the short-term uptake of the radioisotope ^43^K^+^ in *kup9* at different K^+^ concentrations. KUP9 contributes to the distribution of K^+^ in shoots as well as in roots under low K^+^ conditions.

## Results

### Upregulation of *kup9* under low K^+^ conditions

**Fig. 1:**
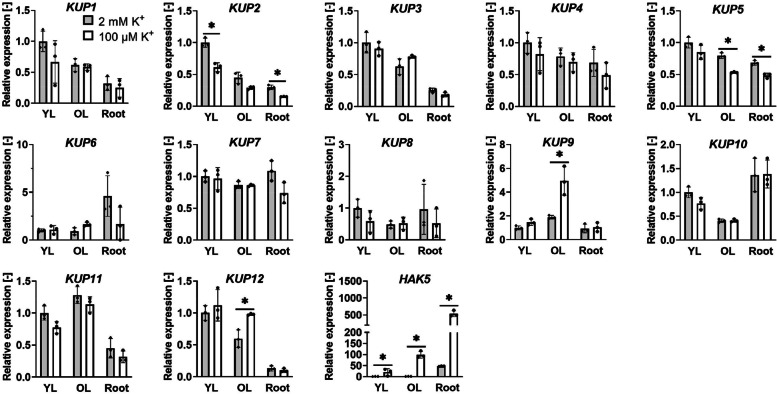
Relative expression of *KUP/HAK/KT* transporters. Plants were grown for 2 weeks in medium containing 2 mM or 100 μM K^+^. Plants were at the same stage when they were dissected into the four youngest leaves (YL), the remaining older leaves (OL) and roots. RT-qPCR was performed to determine the relative levels of the *KUP/HAK/KT* transcripts, using *ubiquitin 10* (*UBQ10*) as the reference gene

While the expression of *KUP/HAK/KT* genes under sufficient K^+^ conditions has been previously determined in Arabidopsis (Ahn et al. 2004), changes in their expression upon K^+^ depletion have not been studied in detail. We isolated total RNA from younger and older leaves and from roots of Col-0 plants grown for 2 weeks on media containing either 2 mM K^+^ (sufficient) or 100 μM K^+^ (low). This study was conducted with media containing NH_4_^+^, these conditions inhibit some K^+^ transport systems including HAK5 (Santa-María et al. 2018). RT-qPCR was performed to determine gene expression of members of the KUP/HAK/KT family with or without K^+^ starvation (Fig. 1). As previously reported, *HAK5* expression was strongly induced in older leaves and roots in response to K^+^ starvation (Ahn et al. 2004). Among the KUP members, *KUP9* (and to a lesser degree *KUP12*) were upregulated in older leaves, while *KUP2* and *KUP5* expression was reduced in both leaves and roots.

### Phenotype of *kup9* under low K^+^ conditions

**Fig. 2:**
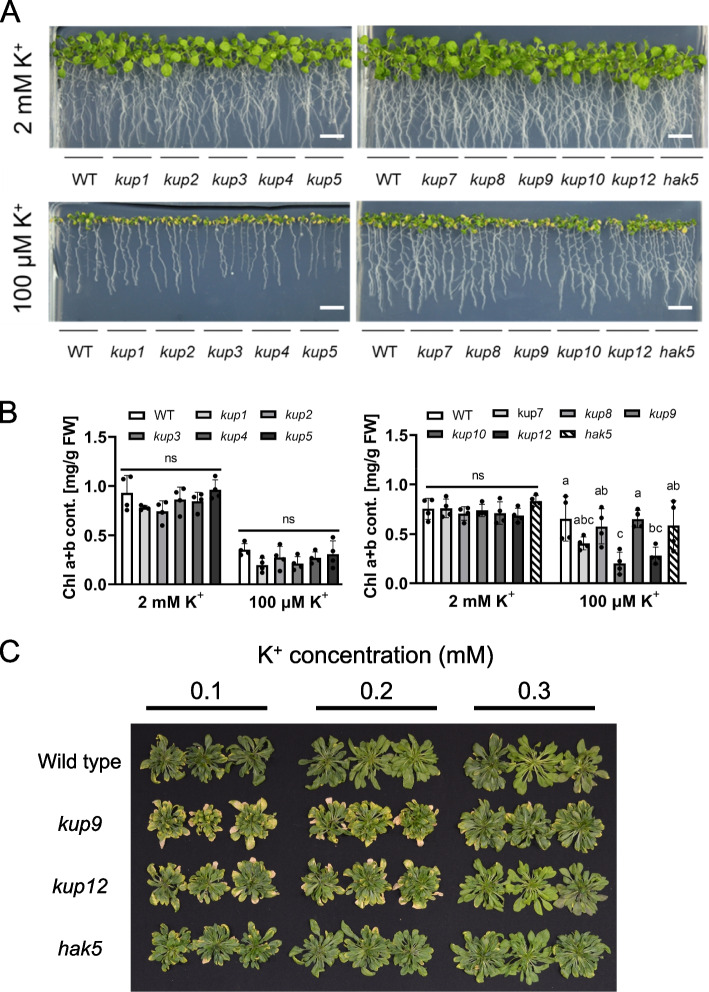
Phenotypes of *kup/hak/kt* mutants under low K^+^ and K^+^ sufficient conditions. **A** Photograph of two-week-old plants grown on medium containing 2 mM (top) or 100 μM K^+^ (bottom). B Chlorophyll content of the shoot tissue of the same plants. Data are shown as mean ± S.D. of *n* = 4 independent plants*.* *P < 0.05, ns, not significant (*P* > 0.05); one-way ANOVA. Scale bar = 1 cm **C** Phenotype during reproductive growth with different amounts of KCl. Representative images of 63-day-old wild-type, *kup9 kup12* and *hak5* plants, grown for the last 30 days on hydroponic medium containing 0.1, 0.2 or 0.3 mM KCl

Next, we investigated the effects of low K^+^ conditions on the phenotypes of KT/KUP/HAK loss-of-function mutants. We obtained homozygous SALK lines for all members of the family, except *kup6* and *kup11* (no lines were available). All *kup* mutant lines were sown on agar plates containing 2 mM K^+^ or 100 μM K^+^, and after 2 weeks of growth the chlorophyll content was determined (Fig. 2). Under low K^+^ conditions, chlorophyll content was reduced in all lines including the wild type. However, both *kup9* and *kup12* showed a stronger decrease in chlorophyll content than the wild type, and *kup9* was the most affected (Fig. 2A and B). In older, hydroponically grown plants, chlorosis was clearly visible at the edges of older rosette leaves of *kup9* at K^+^ concentrations at and below 300 μM, although the overall size of the *kup9* plants was similar to that of the wild type (Fig. 2C). In *kup12* and *hak5* plants, chlorosis at the leaf edges was also observed at or below 200 μM K^+^. These results highlight the contribution of KUP9 in older leaves under K^+^ deficiency.

### Change of *KUP9* expression pattern in response to external K^+^ concentrations

**Fig. 3:**
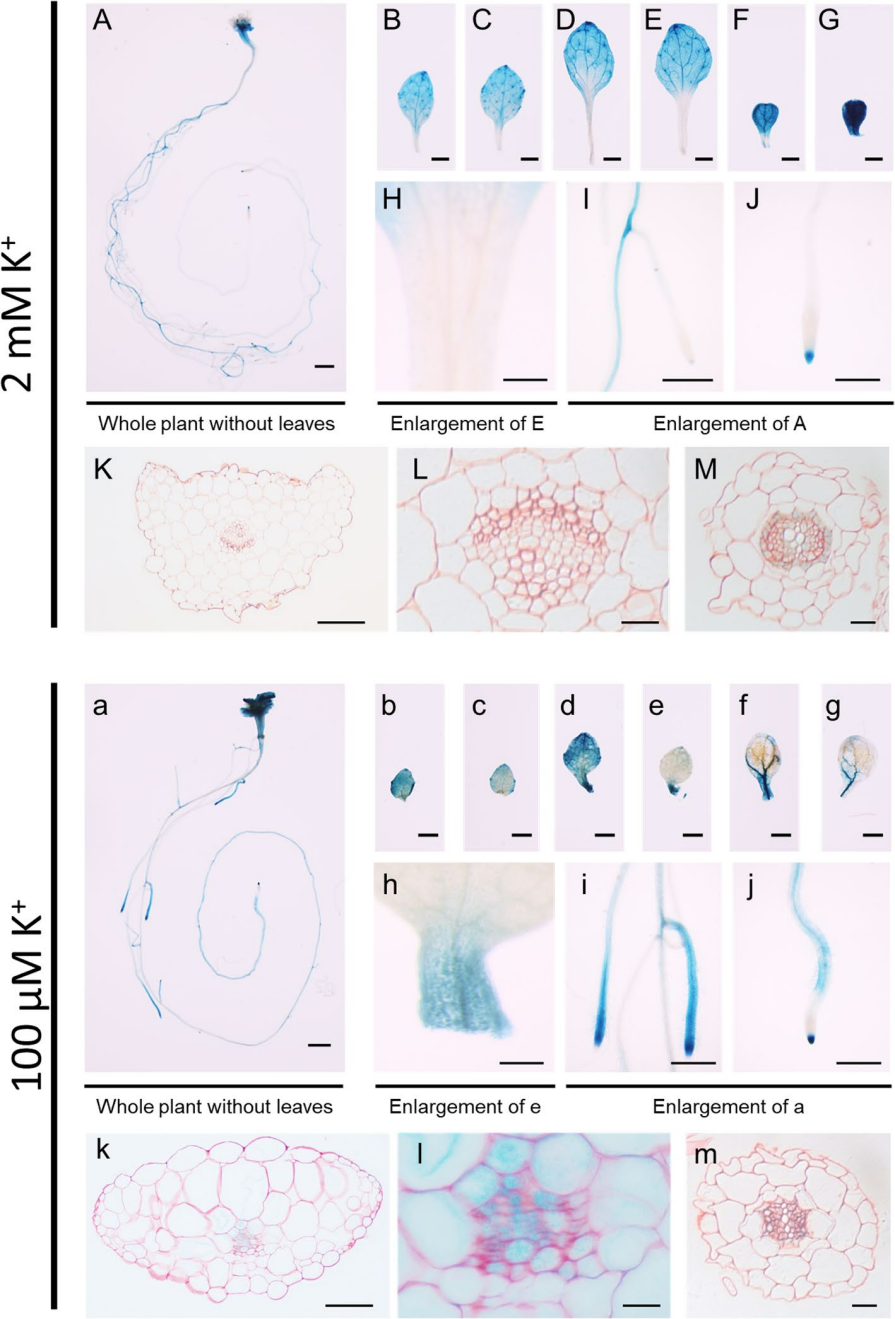
GUS reporter assay of *KUP9* promoter activity *in* Arabidopsis. GUS staining of two-week-old seedlings containing a *KUP9 promoter-GUS* construct grown in medium containing 2 mM K^+^ (A-M) or l00 μM K^+^ (a-m). Whole plant without leaves (A, a), 5th or 6th leaf (B, C, b and c), 3rd or 4th leaf (D, E, d and e), 1st or 2nd leaf (F, G, f and g), petioles (H, h), lateral and primary roots (I, i), and root tip (J, j). Cross-sections of petioles (K, L, k and l) and roots (M and m). L and l are enlarged from K and k. Scale bars represent 1 mm in (A-G and a-g), 0.5 mm in (H-J and h-j), 100 μm in (K, and k) and 20 μm in (L and M, l and m)

To understand the reason for the low-K^+^ sensitivity of *kup9*, we examined the expression pattern of *KUP9* using Col-0 plants transformed with a *KUP9* promoter-GUS construct. Since *KUP9* expression in young seedlings had been determined previously (Zhang et al. 2020; Genies et al. 2021), we performed GUS staining on plants grown for two weeks with either low or sufficient K^+^ (Fig. 3). With sufficient K^+^ supply, *KUP9* expression was observed mainly in the maturation region of the primary root (except for very end of the root tip which was stained under both K^+^ conditions) and in leaves not including petioles. In roots, *KUP9* was mainly expressed in the vasculature (Fig. 3M and m). In contrast, when plants were grown on low K^+^, the expression pattern of *KUP9* shifted to lateral root tips and regions closer to the end of the primary root. The very end of the root tip which was stained under both K^+^ conditions (Zhang et al. 2020; Genies et al. 2021). In leaves, *KUP9* expression shifted to petioles and regions close to them (Fig. 3K, L, k, and l). These results suggested a role of *KUP9* in modulating K^+^ translocation in leaves and roots in response to K^+^ depletion.

### Distribution of K^+^ in *kup9* and *hak5* plants

**Fig. 4:**
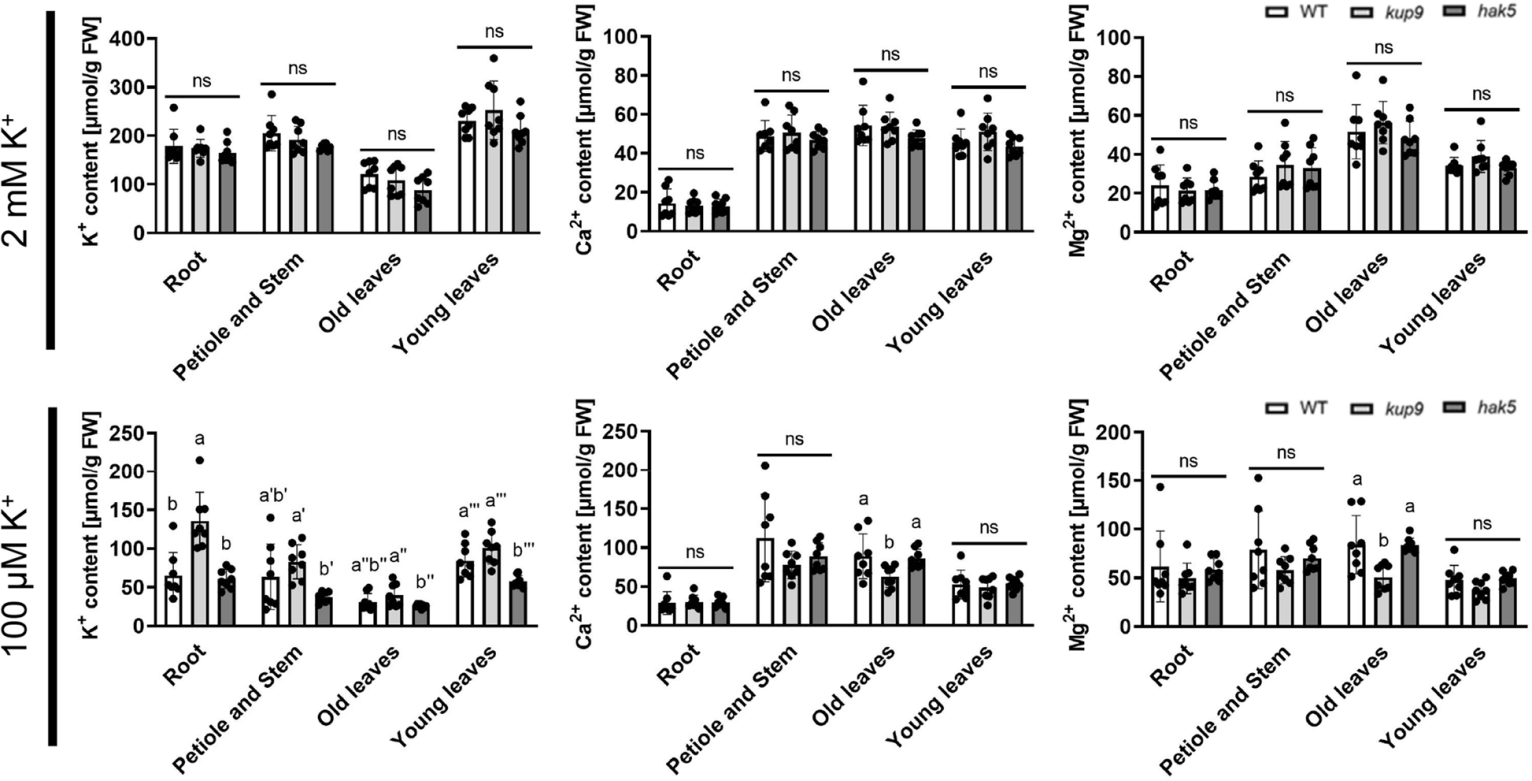
K^+^ accumulation in *kup9* and *hak5*. Plants were grown on synthetic agar medium containing 2 mM K^+^ or l00 μM K^+^ for 2 weeks. K^+^, Ca^2+^ and Mg^2+^ contents of young leaves (four youngest leaves), old leaves (remaining leaves), petioles and stems, and roots of wild-type, *kup9* and *hak5* plants were measured by ICP-OES. Data are shown as mean ± S.D. of *n* = 8 independent samples (four plants in each sample)*. P* < 0.05, *ns*, not significant (*P* > 0.05); one-way ANOVA

**Fig. 5:**
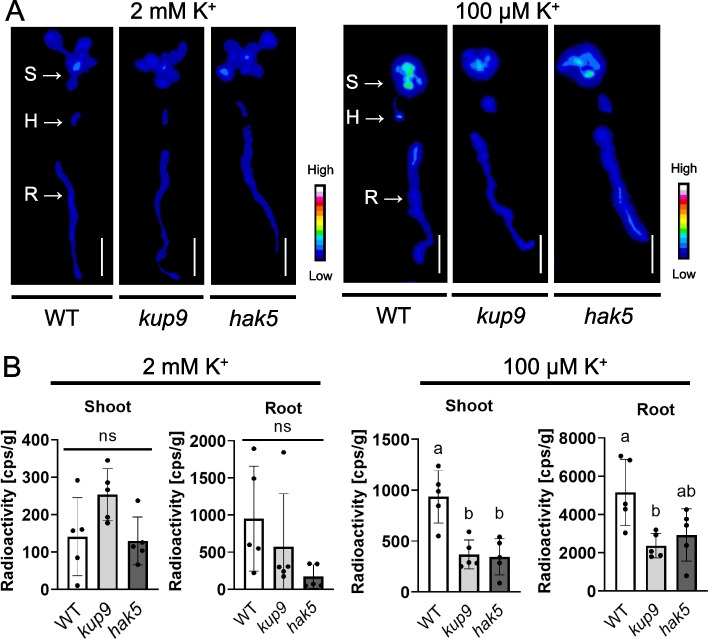
Distribution of ^43^K in *kup9* and *hak5.* **A** Representative autoradiography of the excised shoots (S), hypocotyls (H) and roots (R) of wild-type, *kup9* and *hak5* plants after uptake of ^43^K. Plants were grown on synthetic agar medium containing 2 mM K^+^ (left) or l00 μM K^+^ (right) for 2 weeks before being incubated for 1 h in hydroponic medium containing ^43^K. **B** Quantification ^43^K-uptake into shoots and roots of the plants shown in panel (A). Data are shown as mean ± S.D. of *n* = 5 independent plants. *P* < 0.05, ns, not significant (*P* > 0.05); one-way ANOVA. Scale bars represent 1 cm

To investigate the role of KUP9 in root uptake and translocation of K^+^, we first compared K^+^, Ca^2+^ and Mg^2+^ content of young leaves, old leaves, petioles and stems, and roots of *kup9* and wild type (Fig. 4). As a K^+^ uptake deficient control, *hak5* was used. Under K^+^-sufficient conditions (2 mM), there were no differences in any of the tissues for all three ions, K^+^, Ca^2+^, and Mg^2+^. In contrast, under low K^+^ conditions (100 μM), K^+^ concentrations in the roots of *kup9* were elevated, suggesting that KUP9 contributed to K^+^ translocation from the roots to the shoots. This was supported by the increased expression of *KUP9* in petioles under low K^+^ conditions (Fig. 3). Next, we examined whether there were differences in the short-term K^+^ uptake rate and root-to-shoot translocation speed between wild-type, *kup9* and *hak5* plants by monitoring the uptake of short-lived radio isotopic potassium, ^43^K (Fig. 5). Significant differences were observed between the wild type and *kup9* and *hak5* plants that had been grown under low K^+^ conditions but not between wild type and mutants grown with sufficient K^+^ supply (Fig. 5). In both shoots and roots, K^+^-deficient *kup9* showed less ^43^K accumulation than the wild type. Similar results were obtained for *hak5* shoots and roots. These data suggest that under K^+^ limited conditions KUP9 supports K^+^ uptake in the roots as well as K^+^ translocation to the shoots.

## Discussion

This study revealed the contribution of KUP9 to K^+^ distribution in leaves and roots in response to K^+^ concentration changes. The expression level of *KUP9* increased in older leaves under low K^+^ conditions (Fig. 1) and the expression pattern of *KUP9* changed in leaves and roots in response to the external K^+^ concentrations (Fig. 3). This is consistent with the observed chlorosis in older rosette leaves in *kup9* under low K^+^ conditions (Figs. 2 and 3). K^+^ accumulated in *kup9* roots (Fig. 4) and the short-term K^+^ uptake rate into tissues of *kup9* was reduced, similar to the reduction observed for *hak5* (Fig. 5). Previous reports had focused on the function and role of KUP9 as a K^+^ and Cs^+^ transporter in roots (Adams et al. 2019b; Zhang et al. 2020; Genies et al. 2021). Our study suggests that KUP9 was involved in K^+^ translocation from roots to shoots and K^+^ uptake in roots (Figs. 4 and 5).

KUP/HAK/KT transporters are distinct from K^+^ channels and Trk/Ktr/HKT K^+^ transporters, which share a similar ion conductance profile at their pore regions (Sato et al. 2014). Disruption of *Kup* in *E. coli* inhibits cell growth, which indicates that K^+^ uptake is the primary role of *E. coli* Kup (Tanudjaja et al. 2017). In addition, *E. coli* Kup transports Rb^+^ and Cs^+^, which allows the cells to compensate for K^+^ starvation (Tanudjaja et al. 2017). Arabidopsis KUP9 also transports Cs^+^, similar to other KUP/HAK/KT transporters in Arabidopsis (Qi et al. 2008; Kobayashi et al. 2010; Adams et al. 2013). (Adams et al. 2019b; Zhang et al. 2020; Genies et al. 2021) Moreover, KUP9 also functions as an auxin transporter in the ER membrane in Arabidopsis (Zhang et al. 2020). Our study showed no significant differences in K^+^ content between roots, young and old leaves between the wild type and *kup9,* on sufficient K^+^ media (Fig. 4), which matches previously reported results obtained under similar conditions (Zhang et al. 2020; Genies et al. 2021). In the roots, a significant increase in K^+^ content was found in *kup9*. This result suggests that KUP9 is important for K^+^ translocation from root to shoot (Fig. 4). In contrast, short-term ^43^K absorption was lower in *kup9* than in the wild type (Fig. 5). A similar reduction was found for the ^86^Rb uptake rate in *hak5* (Gierth et al. 2005). The increase in *KUP9* expression in response to K^+^ deficiency also resembled that of *HAK5* in roots.

The results of the *KUP9* promoter-GUS assays (Fig. 3) indicated that the localization of *KUP9* expression in roots and leaves changed in response to the overall K^+^ status of the plant. K^+^-deficiency induced expression of *KUP9* in lateral roots and in regions proximal to the tip of the main root, which might improve K^+^ absorption from the substrate (Adams et al. 2019b; Zhang et al. 2020; Genies et al. 2021). In leaves, *KUP9* expression shifted from the leaf blade to the petiole and areas close to it. Consistent with this, chlorosis occurred at the leaf edges, indicating that those areas were K^+^-deficient (Fig. 3). Leaf chlorosis is a typical symptom of severe K^+^ deficiency. Under K^+^-deficient conditions plants typically redirect the transport of K^+^ from fully expanded leaves or other parts of the shoot towards younger leaves to ensure the supply of these growing tissues (Osmolovskaya et al. 2020). The observed changes in *KUP9* expression in response to K^+^ deficiency are consistent with the hypothesis that KUP9 plays a role in K^+^ translocation in the shoot. The *kup9* mutant was more sensitive to low K^+^-conditions than *hak5* (Fig. 2B and C). Expression of *HAK5* was most highly induced in roots under low K^+^ conditions (100 μM) (Fig. 1), while *KUP9* expression under low K^+^ conditions was induced in leaves, which indicates that KUP9 had functions beyond root K^+^ uptake (Adams et al. 2019b; Zhang et al. 2020; Genies et al. 2021).

KUP9 is located in the ER membrane in roots (Adams et al. 2019b; Zhang et al. 2020; Genies et al. 2021). How does K^+^ move from roots to shoots through KUP9? Recently, KUP9 has been implicated in Cs^+^ efflux from root cells to the apoplast (Adams et al. 2019b; Zhang et al. 2020; Genies et al. 2021). This makes sense if KUP9 is present in the plasma membrane. It is possible that KUP9 changes its intracellular localization depending on the tissue and environmental conditions. Such dual localization exists for KUP4, which localizes to the ER as well as the plasma membrane in certain areas of the root (Rigas et al. 2013; Templalexis et al. 2021). One way to achieve this would be by posttranslational modification. For example, interaction of the *Shaker* K^+^ channel AKT2 with a pair of proteins in the cytosol, calcineurin B-like protein 4 (CBL4) and CBL-interacting protein kinase 6 (CIPK6), promotes the translocation of AKT2 from ER to plasma membrane (Held et al. 2011). This recruitment of AKT2 is dependent on dual lipid modification of CBL4, a common feature among Ca^2+^ dependent phosphorylation modules (Saito et al. 2018). An example of regulation of KUP/HAK/KT transporters by Ca^2+^ dependent phosphorylation is the activation of HAK5-mediated K^+^ uptake via phosphorylation by CIPK23 interacting with CBL1, 8, 9, and 10 (Ragel et al. 2015). It is therefore reasonable to assume that KUP9 could also be regulated by a similar Ca^2+^ dependent phosphorylation system. However, the subcellular localization of KUP9 in shoots has not been determined, and further study will be required to understand the regulation of KUP9 trafficking in leaves.

Data accumulated by several groups have shown that KUP9 transports K^+^ from the ER to the cytoplasm and also promotes Cs^+^ efflux in roots (Adams et al. 2019b; Zhang et al. 2020; Genies et al. 2021). Our study adds new information by showing an involvement of KUP9 in K^+^ transport to the shoots. Together these findings suggest that KUP9 participates in K^+^ and Cs^+^ transport and distribution in plants in cooperation with other transport systems such as HAK5.

## Material and methods

### Plant material and growth

Sterilized seeds were vernalized for 48 h at 4 °C in 1.5-mL centrifuge tubes. Plants were grown on agar medium containing 2 mM or 100 μM K^+^ at 22 °C under 16 h light/8 h dark conditions for 2 weeks. Light intensity was adjusted to around 100 μmolm^− 2^ s^− 1^. The composition of hydroponic medium was 1.25 mM NH_4_H_2_PO_4_, 3 mM Ca(NO_3_)_2_, 1.5 mM MgSO_4_, 100 μM Fe_3_-EDTA, 70 μM H_3_BO_3_, 20 μM MnCl_2_・4H_2_O, 0.3 μM CuSO_4_・5H_2_O, 1 μM ZnSO_4_・7H_2_O, 0.2 μM K_2_MoO_4_, 0.1 μM CoCl_2_・6H_2_O, 0.1% (v/v), Gamborg’s Vitamin Solution (SIGMA-ALDRICH, USA). The pH of the medium was adjusted to pH 5.8 with KOH and then adjusted to 2 mM or 100 μM K^+^ with added KCl. The synthetic agar medium contained the same components as the hydroponic medium plus 0.8% agar and 0.1% sucrose. To create K-limited conditions during reproductive growth, plants were first grown on 1/2 MS agar medium for 25 d and then transferred to liquid medium (2 mM MgSO_4_・7H_2_O, 2 mM NaH_2_PO_4_, 2 mM NH_4_NO_3_, 2 mM Ca(NO_3_)_2_, 0.05 mM Fe(III)EDTA and 2 μM KI) containing 2 mM KCl for 8 d to acclimate to hydroponic culture. Then, plants were transferred to liquid medium containing 0.1, 0.2 or 0.3 mM KCl and grown for 30 d at 23 °C under 8 h light/16 h dark conditions. When bolting occurred, the stems were removed to encourage the growth of leaves. The *Arabidopsis* Columbia ecotype (Col-0) and T-DNA insertion lines including *kup1* (SALK_051343), *kup2* (SALK_097636), *kup3* (SALK_002622), *kup4* (SALK_043791), *kup5* (SALK_072850), *kup7* (SALK_206158), *kup8* (SALK_041357), *kup9* (SALK_108080), *kup10* (SALK_072956), *kup12* (SALK_083613) and *hak5* (SALK_130604) were obtained from the Arabidopsis Biological Resource Center (ABRC).

### qPCR analysis

Total RNA was extracted from the younger four leaves (including meristematic tissue), the rest of older the leaves, and roots of 2-week-old plants using TRI REAGENT (Molecular Research Center, lnc). ReverTra Ace® qPCR RT Master Mix with gDNA Remover (TOYOBO CO., LTD) was used for genomic DNA removal and cDNA synthesis. The reverse transcription reaction protocol consisted of 37 °C for 15 min, 50 °C for 5 min, and 98 °C for 5 min. qPCR was performed using a StepOnePlus Real-Time PCR System (Applied Biosystems, USA). Expression level is shown as values relative to the expression level of the reference gene of Ubiquitin 10 (UBQ10) (Tong et al. 2009). For each gene, the relative expression under various conditions (2 mM K^+^ and 100 μM K^+^) and in different tissues (the four youngest leaves, the remaining older leaves and roots) was then calculated in relation to its expression level in younger leaves with 2 mM K^+^.

### Chlorophyll content

Shoot tissue was sampled in 1.5 ml tubes and fresh weight was determined. 80% (v/v) of cold acetone solution was added and left overnight at 4 °C in dark. Samples were then centrifuged at 4 °C, 15,000 rpm for 15 minutes, and absorbance of the supernatant solution was measured at 646 nm, 663 nm, and 750 nm with a double-beam spectrophotometer (HITACHI UH5300). The amount of chlorophyll in the aboveground tissue was determined from the concentration of chlorophyll in the solution, which was calculated from the following formula using the measured values.

1 $$Chl\ a+b\ \left[\mu M\right]=19.54\times \left({A}_{646}-{A}_{750}\right)+8.29\times \left({A}_{663}-{A}_{750}\right)$$

### GUS staining

Arabidopsis *kup9* promoter sequence (2864 bp) was amplified by PCR using a pair of primers, 5′-TGATTACGCCAAGCTTCTGCATCATCAAACAGAG-3′ and 5′- AGGGACTGACCACCCGGGTTTTGTAACAAAAGAACT-3′ and cloned into HindIII and Smal-digested pBI101 (provided by Kenzo Nakamura) using the In-Fusion® HD cloning kit (Takara Bio USA, Inc.) (Ohta et al. 1990). The final construct consisting of the *β*-glucuronidase (*GUS*) gene under control of the AtKUP9 promoter (*ProAtKUP9:GUS*) was introduced into *Arabidopsis thaliana* Col-0 via Agrobacterium-mediated transformation. Seeds of *Arabidopsis thaliana* expressing *GUS* gene were grown on solid medium containing 2 mM or 100 μM K^+^ for two weeks. The ProAtKUP9:GUS plants were analyzed for GUS expression according to standard procedures (Jefferson et al. 1987). For each treatment whole plants were incubated in staining solution containing 0.5 mM X-Gluc (5-bromo-4-chloro-3-indolyl-*β*-D-glucuronide) at 37 °C for 3–4 h and destained in 70% ethanol. To prepare tissue sections, GUS-stained plants were dehydrated with 80%, 90% and 100% ethanol for one day each, and then fixed in Technovit 7100 (Heraeus Kulzer, Tokyo, Japan). The tissue was sliced into 6 μm sections using a Leica RM2145 microtome (Leica, Wetzlar, Germany). Sections were stained with 0.01% neutral red (FUJIFILM Wako Chemicals, Japan) for 1 min and then rinsed twice with water. After drying the samples, they were covered with Entellan® new (FUJIFILM Wako Chemicals, Japan) and were examined under a microscope.

### K^+^ content determination

Plants grown for 2 weeks with 2 mM or 100 μM K^+^ were divided into the younger four leaves (including meristematic tissue), the remaining older leaves, the petioles and stems, and roots. Plant materials from four individual plants were placed into 1.5 ml tubes and dried at 60 °C for at least 24 h. Then 400 μl of nitric acid was added to each sample, and were left to digest overnight or longer. The nitric acid extracts were diluted 50-fold with MiliQ water and the amount of K^+^, Ca^2+^, and Mg^2+^ was determined with an inductively coupled plasma optical emission spectrometer; ICP-OES (iCAP 6500; Thermo Fisher Scientific, USA).

### Production of ^43^K^+^

Potassium-43 (^43^K^+^) was produced in the ^nat^Ca(γ,pxn) reactions using an electron linear accelerator at the Research Center for Electron Photon Science at Tohoku University. The calcium oxide was sealed in quartz tubes and irradiated with bremsstrahlung for several hours under water cooling. Produced ^43^K^+^ was separated from the calcium oxide by an oxalate precipitation method and purified by cation exchange chromatography. The ^43^K^+^ tracer was dried and dissolved in hydroponic medium containing 2 mM K^+^. For the ^43^K^+^ absorption experiments, plants were grown on 2 mM or 100 μM K^+^ synthetic agar medium without tracer for 2 weeks. Then plants were incubated in hydroponic medium (10 ml) supplemented with 2 MBq of ^43^K^+^ for 1 h and potassium absorption was determined.

### Image analysis

After 1 h, the plants were washed twice with hydroponic medium containing 2 mM K^+^, and dissected with surgical scissors into shoots, hypocotyls and roots. The plant material was put onto imaging plates (Fuji Film, Japan) and exposed for 3–5 h at 4 °C. The imaging plates were scanned in a Typhoon FLA 9500 laser scanner (GE Healthcare Japan K.K., Japan). The plant material was then transferred into individual tubes and the weight was determined.

### Quantitative analysis by γ-ray spectrometry

After the image analysis, the plant parts were analyzed for the amount of ^43^K radioactivity via g-spectrometry with a germanium semiconductor detector of 35% relative efficiency. For measurements, samples were placed at 2 mm from the surface of the detector, which was housed in a lead-shield box. The radioactivity of the ^43^K was determined from the peak area at 373 keV.

## Abbreviations

HAK/KUP/KT: High-Affinity K^+^ Transporter/K^+^ Uptake Permease/K^+^ Transporter
CPAs: Cation/Proton Antiporters
CBL: Calcineurin B-Like
CIPK: CBL-Interacting Protein Kinase
CHX: Cation/H^+^ Exchanger
CNGC: Cyclic Nucleotide Gated Channel
IAA: Indole-3-Acetic Acid
ER: Endoplasmic Reticulum
GUS: *β*-Glucuronidase
UBQ: Ubiquitin
qPCR: Quantitative PCR
